# The Effect of Adding Grape Seed Powder Modified by an Alternating Current Electric Field on the Properties of Chicken Myofibrillar Protein Gel

**DOI:** 10.3390/foods15132299

**Published:** 2026-06-26

**Authors:** Xingyu Liu, Zhongwen Cao, Guangyi Bie, Xiangren Meng

**Affiliations:** 1 School of Tourism and Cuisine, Yangzhou University, Yangzhou 225127, China; 2 Institution of Catering Culture, Yangzhou University, Yangzhou 225127, China; 3 Key Laboratory of Chinese Cuisine Intangible Cultural Heritage Technology Inheritance, Ministry of Culture and Tourism, Yangzhou 225127, China

**Keywords:** alternating electric field, modified grape seed powder, gel

## Abstract

This study examined the impact of varying addition levels (0%, 0.5%, 1%, 2%, and 4%) of electro-processed grape seed powder on the physicochemical properties and gel features of chicken myofibrillar protein. The incorporation of modified grape seed powder (MGSP) with an alternating electric field can strengthen the structure of myofibrillar protein (MP) gel, as well as improve its water retention capacity and solubility to a degree. Moreover, as the amount of addition increases, the gel’s hydrophobicity decreases and the number of carbonyl groups decreases, but the antioxidant activity improves. Ionic bonds and disulphide bonds are predominant intermolecular forces in the MP gel. MGSP can induce alterations in protein conformation and enhances the development of a dense and homogeneous gel network. Overall, when the addition amount is 2–4%, the gel characteristics reach optimal conditions. This study offers a crucial informational foundation for the utilization of plant-based materials altered by alternating electric fields and composite gels in meat products.

## 1. Introduction

As civilization progresses, an increasing number of individuals focus on the nutrition and functionality of food [1]. Meat products frequently pose issues due to their elevated fat and cholesterol levels, as well as their inadequate antioxidant properties. Individuals choose chicken due to its high protein content and affordability, which accounts for its popularity in the fitness sector. Muscle fiber protein (MP) gel constitutes the essential component of all beef products. Modifications in the structure of myofibrillar proteins may influence their water-holding capacity, emulsifying properties, and antioxidant attributes, hence altering the quality of meat products [2]. Optimal gel characteristics are essential for the manufacture and storage stability of meat products, as well as for satisfying consumer quality expectations. However, chicken lacks elastic and antioxidant characteristics. Suboptimal yield and texture tailored for industrial production and customer specifications. Consequently, investigations into the application of natural active substances to enhance meat quality and product qualities have markedly intensified [3]. Grape seeds comprise a complex composition of 40% fiber, 16% oil, 11% protein, and 7% phenolic chemicals [4]. It is an economical source of antioxidants owing to its elevated phenolic compounds [5] and its capacity to scavenge free radicals [6], which is beneficial for human health. In Japan, grape seed extract is acknowledged as a permissible food ingredient [7]. Grape seed polyphenols can induce the aggregation of oxidized proteins in meat and promote the formation of insoluble protein aggregates [8]. Yang et al. [9] demonstrated that the inclusion of grape seed polyphenols can reduce the formation of carbonyl and free amino groups and restrict the exposure of hydrophobic groups, thus enhancing the crosslinking among proteins and ultimately improving the structural properties of the MP gel.

Alternating current electric field (ACEF) technology is extensively utilized in food preservation, sterilization, and freezing because of its cost-effectiveness and energy efficiency. The ACEF treatment has been shown to alter the structure of peanuts and reduce the generation of undesirable volatile compounds [10]. The ACEF treatment can induce the absorption or aggregation of the polar groups in protein molecules, disrupting intermolecular interactions like hydrophobic forces, thus altering protein conformation and enhancing material quality and flavor [11].

Previous studies have shown that ACEF can significantly alter the physico-chemical properties of plant-derived materials, improve their dispersion stability, and increase the accessibility of phenolic functional groups, hence enhancing antioxidant activity and functional characteristics. These modifications may enhance the interaction between grape seed powder and protein molecules, hence increasing its effectiveness as a functional ingredient in meat systems [12]. Currently, there is inadequate research about the mechanism via which GSP affects the properties of animal protein gels. This study aims to investigate the impact of different concentrations (0%, 0.5%, 1%, 2%, and 4%) of modified grape seed powder on the elasticity, water-binding capacity, antioxidant properties, hardness, and structure of chicken myofibrillar protein gels, as well as to elucidate the mechanism by which grape seed powder influences these properties. The expected outcome will provide significant theoretical insights for the application of the composite gel in meat products in the food industry.

## 2. Materials and Methods

### 2.1. Materials

GSP was obtained from Anhui, China. Fresh chicken breasts were procured at Darunfa Supermarket in Yangzhou, China. All chemical reagents employed in this study procured from Basite Chemical Co., Ltd. (Nanjing, China).

### 2.2. Alternating Electric Field Treatment

In our previous study [12], it was found that the ACEF significantly improved the functional properties of GSP, with the best effect at 80 V/cm. Therefore, GSP was treated at 80 V/cm for 10 min at a frequency of 400 Hz. The experiment was carried out with a laboratory-built device for alternating electric fields. The apparatus consisted of a quartz heating chamber with two stainless steel electrode plates, an AC frequency converter power supply (ANJ13-1 kilowatt, Anya Power Supply Company, Suzhou, China), and a control computer [12]. The treated GSP was freeze-dried using a freeze-drying machine (SCI ENTZ-18 N, Sunlight Huan Optoelectronic Devices Manufacturing Co., Ltd., Ningbo, China) to obtain MGSP. It was stored in a constant-temperature drying dish at room temperature.

### 2.3. Preparation of MP Gels

The operation was performed with minor modifications of the approach previously reported [13]. Dice the chicken breast into small fragments, combine them with pre-cooled phosphate-buffered saline (PBS) (1:4, *w*/*v*), and subsequently homogenize using a homogenizer (TGL 16M, Luxiangyi Centrifuge Instrument Co., Ltd., Shanghai, China) at a speed of 9000 revolutions per minute for 20 s. The homogenate was centrifuged at 3000× *g* for 10 min by a centrifuge (FJ200, Shanghai Huexi Industrial Co., Ltd., Shanghai, China). The resultant precipitate was rinsed four times with PBS and centrifuged at each washing step. Thereafter, the precipitate was rinsed with a 0.1 mol/L NaCl solution employing the identical procedure. The resultant precipitate was the myosin protein (MP) solution, which was preserved at 4 ± 2 °C. The prepared MP was added with 0%, 0.5%, 1%, 2%, and 4% MGSP and was named MGSP-0, MGSP-0.5, MGSP-1, MGSP-2, and MGSP-4, respectively.

### 2.4. Color Difference and Whiteness

The color measurement was modified to a certain degree in accordance with the methodology of Shi et al. [14]. The apparatus was calibrated with a reference plate, and the gels were sectioned into 1.5 × 1.5 × 1 cm cubes and assessed using a spectrophotometer (CM-700d, Konica Minolta, Tokyo, Japan). Record the values of L*, a*, and b*, and then calculate the whiteness based on these three values.

(1) $$Whiteness=100-\sqrt{\begin{array}{c} {\left(100-L^{*}\right)}^{2}+{a^{*}}^{2}+{b^{*}}^{2} \end{array}}$$

### 2.5. Water Holding Capacity

Modifications were implemented in the relevant sections, as per the preceding methodology [15]. Record the total weight of 1 g of the gel and the test tube (W1). Centrifuge at 8400 rmp for 12 min. Post-centrifugation, decant the produced water and utilize absorbent paper to collect the residual moisture. Reweigh the residual gel and the tube (W2). Determine the water-holding capacity using Equation (2).

(2) WHC (%)=W2/W1×100

### 2.6. Texture Properties of Gels

The procedure for assessing gel strength was somewhat altered as outlined by Lv et al. [16]. A texture analyzer (TA-3000, Saicheng Electronic Technology Co., Ltd., Jinan, China) equipped with a P/0.5 probe was used to assess the gel samples’ elastic properties. The following were the program’s parameters: pre-test velocity: 1.0 mm/s, compression distance: half of the sample height, test speed: 1.0 mm/s, post-test speed: 1.0 mm/s, and contact force: 5.0 g.

### 2.7. Protein Solubility

The protein solubility assessment method has experienced some changes in comparison to the previously documented method [17]. The MP solution was diluted (2 mg/mL) with PBS (pH 6.5) and subsequently vortexed for 20 s. Subject the solution to centrifugation at 8000 rmp for 12 min, followed by analysis of the supernatant. The quantities of soluble protein (C1) and total protein (C2) were quantified by colorimetric analysis. The protein solubility was determined utilizing the subsequent formula:

(3) $$Protein\;solubility\;(\%)=\frac{C_{1}}{C_{2}}\times 100$$

### 2.8. Surface Hydrophobicity

A minor alteration was implemented in the research methodology of Nie et al. [18] to assess hydrophobicity. The initial MP solution was diluted to a target concentration of 5 mg/mL using a PBS solution. The diluent was combined with bromophenol blue (BPB) solution (5:1, *v*/*v*), while an equivalent volume of phosphate-buffered solution served as the control group. All sample groups underwent centrifugation at 8000 rmp for 10 min, after which the supernatant was diluted tenfold. The absorbance levels of the control group (A0) and the experimental group (A) were ultimately quantified. Subsequently, compute the surface hydrophobicity:

(4) $$BPB\;bound\;(\mu g)=200\;\mu g\times \left(A_{0}-A\right)/A_{0}$$

### 2.9. Free Sulfhydryl Groups

Thoroughly combine 9 mL of PBS with 1 mL of MP solution (2 mg/mL). Combine the sample mixture with 0.1% of 5,5’-dithiobis(2-nitrophenol) (10:1, *v*/*v*). The sample is incubated at 40 °C for 35 min, using a wavelength of 412 nm to measure the absorbance. A phosphate-buffered solution devoid of MP serves as a blank control [19].

### 2.10. Total Carbonyl Content

Combine 1 mL of MP solution with a protein concentration of 2 mg/mL and 2,4-dinitrophenylhydrazine (DNPH) in a light-excluded environment for 1 h. Subsequently, introduce 20% trichloroacetic acid to halt the process. Utilize a centrifuge at 10,000 rpm for 10 min to get the precipitate. Wash it multiple times with a solution of ethyl acetate combined in a volumetric ratio until the color is entirely removed. Dissolve the precipitate in 1 mL of guanidine hydrochloride solution (6M) and subsequently agitate uniformly at 37 °C for 35 min. Following centrifugation, extract the supernatant and assess the absorbance at 370 nm [20].

### 2.11. DPPH Free Radical Scavenging

The *DPPH free radical scavenging* was assessed using the method of Zeng et al. [21], with some changes. The combination underwent centrifugation at 8000 revolutions per minute for 20 min, after which the supernatant was harvested. Three groups were established: A1: 2 mL of the supernatant combined with a 2,2-diphenyl-1-pyridinium hydroxide (DPPH) ethanol solution; A2: 2 mL of the supernatant mixed with ethanol; A3: 2 mL of deionized water combined with the DPPH ethanol solution. Following a 40 min incubation of all samples in darkness, the absorbance was assessed at a wavelength of 517 nm. The DPPH radical scavenging activity was determined using the subsequent formula:

(5) $$DPPH\;clearance\;rate\;(\%)=\frac{A_{3}-A_{1}+A_{2}}{A_{3}}\times 100$$

### 2.12. Molecular Interactions

Disperse 1 g of the gel sample into 10 mL of the extraction solution, comprising 0.05 M NaCl (S1), 0.6 M NaCl (S2), 0.6 M NaCl and 1.5 M urea (S3), 0.6 M NaCl and 8 M urea (S4), and 5 M β-mercaptoethanol (S5). The samples underwent homogenization for 1.5 h. Subsequently, the supernatant was centrifuged at 8500 rmp for 15 min, and the protein concentration in the supernatant was quantified using the Bradford method. The variations in protein concentrations across the five treatment groups facilitated the calculation of electrostatic interaction, hydrogen bonds, hydrophobic effect, and disulphide linkage [22].

### 2.13. Rheological Analysis

Deposit 2 g of the MP solution onto a stainless steel plate. Administer silicone oil around the specimen to inhibit water evaporation. The separation between the two plates is 1 mm. The sample was heated at a consistent pace from 20 °C to 80 °C over a duration of 30 min. The constant strain was established at 2%, while the frequency was designated at 0.1 Hz. The storage modulus (G’) and the loss modulus (G”) were also measured [23].

### 2.14. Analysis of Fourier Transform Infrared (FTIR) Spectroscopy

The samples positioned on the ATR crystal surface were examined with the PerkinElmer Frontier FTIR spectrometer (Cary 610/670, Varian Medical Systems, Palo Alto, CA, USA), with the scanning range established at 4000–400 cm^−1^.

### 2.15. SEM

The cross-sectional slices of the freeze-dried materials were examined with a scanning electron microscope (GeminiSEM 300, Carl Zeiss, Jena, Germany) at an accelerating voltage of 5.0 kV and a magnification of 100×.

### 2.16. Data Analysis

The results of all experiments, after being repeated three times, are presented as the mean value ± standard deviation. All charts and statistical analysis were generated using Origin 2024 software. Significance was assessed using ANOVA variance analysis, with *p* < 0.05 denoting significance.

## 3. Results

### 3.1. Color Difference and Whiteness

Table 1 shows the L*, a*, b*, and white value of five groups of chicken muscle fibrin protein gels. The incorporation of MGSP will affect the coloration of the protein gel (*p* < 0.05). The a* value of the gel is directly proportional to the MGSP concentration, whereas the L*, b*, and whiteness values are inversely proportional. This was because MGSP contains brown pigments, which altered the gel’s color characteristics [24]. The same result was also observed in the sheep muscle fibrin protein gel with grape seed added [8]. The results above indicate that MGSP can, to some extent, alter the color of the protein gel. Therefore, the additional amount of MGSP should be reasonably controlled while improving the quality.

### 3.2. WHC

**Figure 1:**
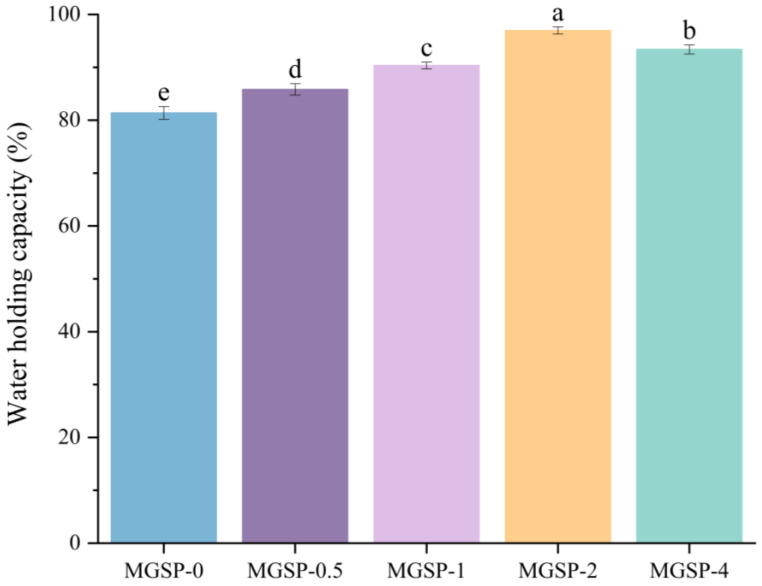
The impact of the quantity of MGSP added on the water holding capacity of MP gels. a–e: Distinct lowercase letters signify substantial changes in values among groups (*p* < 0.05). Error bars indicate the standard deviation.

Water retention capacity is a widely utilized metric to assess the water content in the protein gel network of muscle fibers, indicating the spatial configuration of the protein gel [25]. It is frequently utilized for the objective evaluation of the production level and quality of meat and meat products [26]. Figure 1 illustrates the gel’s water retention capability. The bar chart indicates an improvement in the water-holding capacity of the muscle fiber protein gel incorporating modified grape seed powder (MGSP). As the quantity of additive increases, the water retention capacity escalates. At an added amount of 2%, the water-holding capacity reaches its peak, exhibiting an increase of 11.8%. The enhancement of its WHC may be ascribed to the dietary fiber included in grape seed powder. Suitable dietary fiber can enhance the gel’s network architecture, increasing its resistance to centrifugal force [15]. Conversely, when the added amount rises to 4%, the water-holding capacity diminishes. Excessive MGSP will markedly diminish the concentration of sulfhydryl groups in the gel, compromising the integrity of the three-dimensional gel network structure and consequently resulting in a reduction in WHC [27]. The findings indicate that including a composite dietary fiber during the heat gelation process enhances MP cross-linking and markedly increases the WHC of the resultant gel structure [28]. Related investigations indicated that as the quantity added increased, the water retention capacity initially ascended and subsequently declined, exhibiting a similar pattern [29].

### 3.3. Texture Properties (TPA) of Gels

The mechanical properties of MP gels are often assessed using the measure of gel strength [30]. Table 2 illustrates the impact of differing MGSP content on the textural properties of the composite gel. MGSP markedly improved the elasticity, hardness, adhesion, and gummines characteristics of the composite gel (*p* < 0.05). The most notable enhancement was observed with MGSP-2, which, at a 2% incorporation rate, elevated the hardness and gumminess of the gel by 53.88% and 78.23%, respectively. This may result from insoluble dietary fibers in MGSP occupying vacancies in the gel network, thus reinforcing its structure [31]. Nevertheless, the gel’s springiness and cohesiveness diminished in comparison to the control group. This may result from GSP’s shielding effect on the reactive functional groups of proteins, which impedes protein cross-linking and consequently diminishes the gel’s springiness and cohesiveness [2]. This aligns with the research findings about the impact of grape seed powder on the structural alterations of sausages [32]. In conclusion, MGSP can enhance the strength of protein gels; however, it is essential to regulate the quantity added judiciously.

### 3.4. Protein Solubility

**Figure 2:**
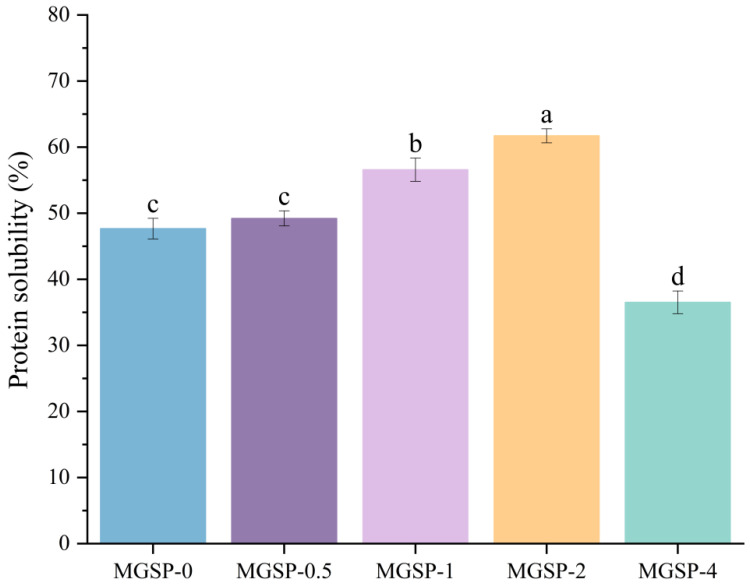
The impact of the quantity of MGSP added on the protein solubility of MP gels. a–d: Distinct lowercase letters signify substantial changes in values among groups (*p* < 0.05). Error bars indicate the standard deviation.

**Figure 3:**
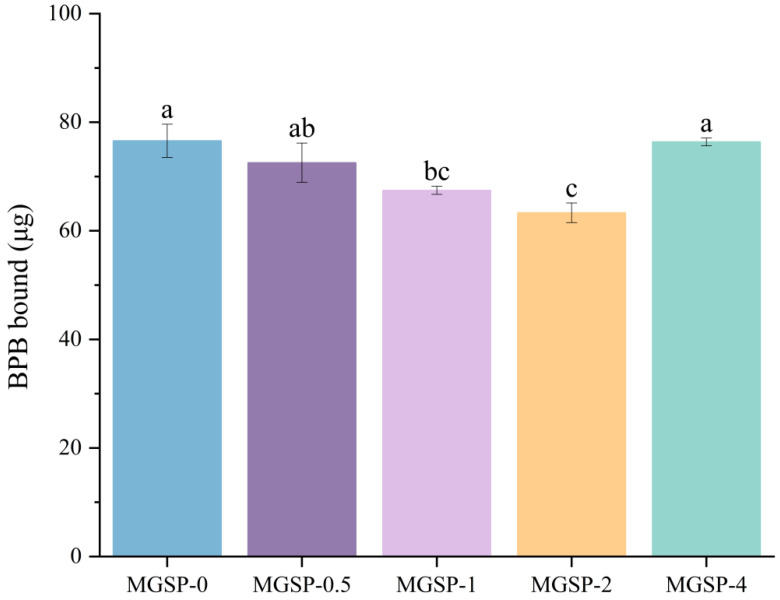
The impact of the quantity of MGSP added on the hydrophobicity of MP gels. a–c: Distinct lowercase letters signify substantial changes in values among groups (*p* < 0.05). Error bars indicate the standard deviation.

Protein solubility serves as the most suitable metric for assessing protein denaturation and aggregation [33] and is intricately linked to functional attributes such as emulsification, dispersion, and gelation [34]. Figure 2 illustrates the protein solubility. As the concentration of MGSP rises, solubility progressively enhances. With an addition of 2%, solubility increases by 29.47%. This may be ascribed to the capacity of the dietary fiber in grape seed powder to impede protein aggregation [35]. The enhancement of solubility results in a greater number of proteins engaging in gel formation, thus augmenting the gel’s WHC. Moreover, it signifies an improved stability of polyacrylate (MP) and a decrease in the exposure of hydrophobic groups. Hydrophobic groups diminish the attraction between polyacrylate and water molecules [36]. The surface hydrophobicity results of MP (Figure 3) corroborate this fact. However, as the additive quantity surpasses 4%, solubility diminishes. This may result from the interaction between elevated phenolic chemicals in MGSP and microparticle proteins, which diminishes protein solubility [37,38]. Moreover, the establishment of non-covalent or covalent connections between polyphenols and particulate proteins will lead to the creation of protein-polyphenol complexes, thus diminishing their solubility [39].

### 3.5. Surface Hydrophobicity

The hydrophobicity of proteins is typically indicative of their binding affinity with BPB [40]. The augmentation of surface hydrophobicity often signifies an elevation in the accessibility of hydrophobic groups and alterations in the protein conformation [41]. Figure 3 illustrates the outcomes of hydrophobicity. In comparison to the untreated MP gel, the surface hydrophobicity of myosin filaments diminished following the incorporation of MGSP, with a minimum decrease of 17.3%. This suggests that MGSP may create polymers via protein–protein interactions, thus preventing protein unfolding, concealing or shielding surface hydrophobic groups, diminishing their exposure, and thereby reducing surface hydrophobicity [42]. Research indicates that an augmentation in protein–protein interactions may result in the degradation of specific non-polar amino acids, thereby diminishing the surface hydrophobicity of proteins [43]. Furthermore, MGSP primarily consists of insoluble dietary fibers. Lu et al. [44] revealed that insoluble dietary fibers improve the gel properties of myofibrillar protein and reduce surface hydrophobicity. As the quantity of addition escalates, hydrophobicity also rises, likely due to the creation of bigger aggregates resulting from interactions between proteins and MGSP, which enhances surface hydrophobicity [18].

### 3.6. Total Carbonyl Groups

**Figure 4:**
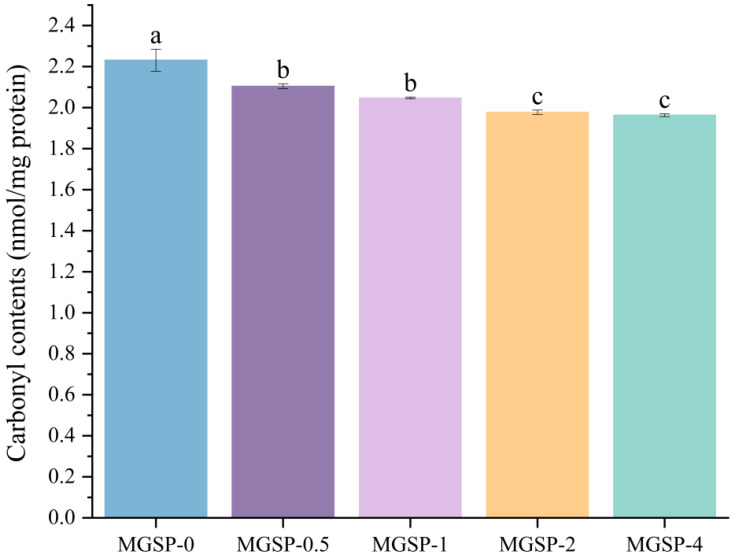
The impact of the quantity of MGSP added on the carbonyl contents of MP gels. a–c: Distinct lowercase letters signify substantial changes in values among groups (*p* < 0.05). Error bars indicate the standard deviation.

The presence of carbonyl groups may indicate the buildup of oxidative products produced by protein side chains [45]. The impact of MGSP on protein oxidative molecular damage was evaluated by measuring the carbonyl group level in the MP solution. The concentration of carbonyl groups was noted to diminish to different extents in the MP solution containing MGSP (Figure 4). This suggests that protein oxidation was suppressed, particularly at an addition concentration of 2%, where an 11.37% reduction was noted. The free radical scavenging capacity of GSP somewhat mitigated the oxidation of protein side chains, thereby diminishing the levels of groups that transform amino acid residues into carbonyls [8]. Overall, it demonstrates that MGSP effectively suppresses the production of carbonyl groups.

### 3.7. Free Sulfhydryl Groups (R-SH)

**Figure 5:**
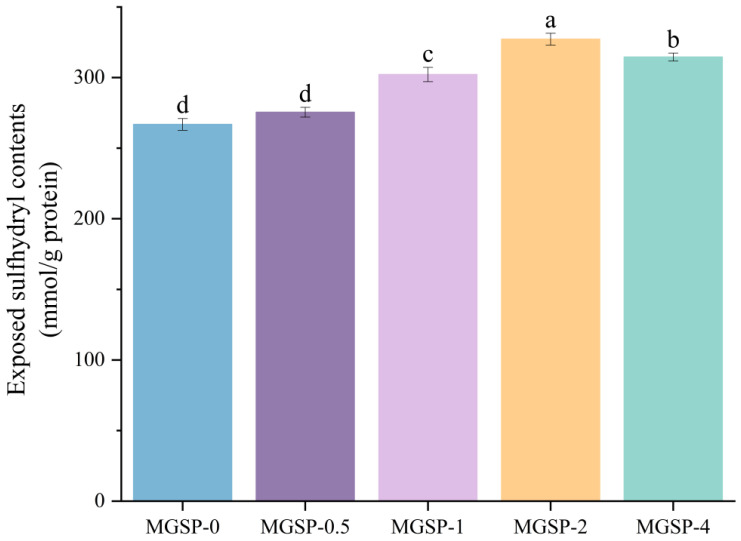
The impact of the quantity of MGSP added on the free thiol groups of MP gels. a–d: Distinct lowercase letters signify substantial changes in values among groups (*p* < 0.05). Error bars indicate the standard deviation.

The thiol group, the most reactive functional group on protein side chains, readily forms a disulfide bond via a dehydrogenation process. A reduction in sulfhydryl group content typically signifies that the protein has experienced oxidation [46]. The concentration of *R-SH* in the MP exhibited varied degrees of increase with alterations in MGSP concentration (Figure 5). This suggests that the oxidative damage to myofibrillar protein with additional MGSP is reduced, particularly at a concentration of 2%. This may result from an enhancement of antioxidant active compounds in grape seed powder following modification by an alternating-current electric field. These components can safeguard proteins against the development of carbonyl groups [47]. MGSP is abundant in polyphenols, which safeguard the sulfhydryl groups in myofibrillar protein, diminish the production of disulphide bonds, and mitigate the erratic cross-linking in oxidized myosin [48]. Research indicates that polyphenols can mitigate the depletion of sulfhydryl groups in chicken gel during manufacture and storage due to protein oxidation [49]. The reduction at 4% concentration may diminish water-holding capacity, in accordance with the alteration depicted in Figure 1. This may occur from the increased concentration of grape seed powder particles inside the gel network, leading to inconsistent connections between protein fibrils and promoting oxidative processes [50].

### 3.8. DPPH Radical Scavenging Ability

**Figure 6:**
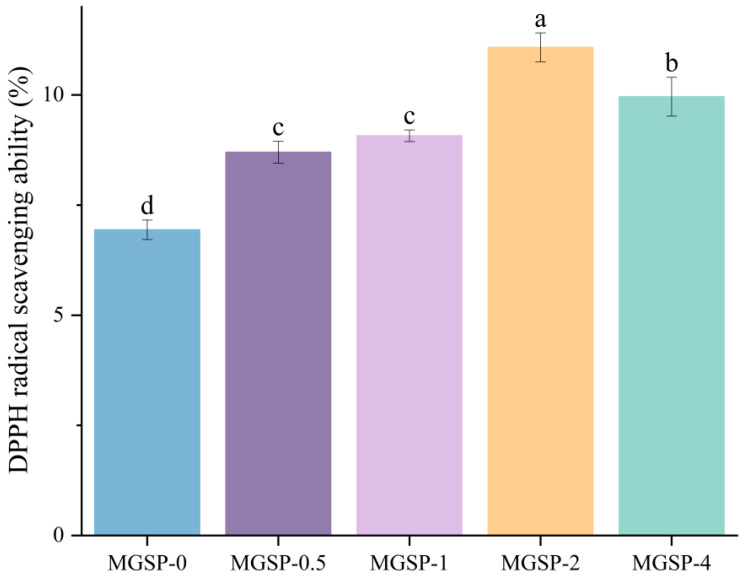
The impact of the quantity of MGSP added on the DPPH free radical scavenging ability of MP gels. a–d: Distinct lowercase letters signify substantial changes in values among groups (*p* < 0.05). Error bars indicate the standard deviation.

The DPPH method is widely utilized to evaluate the antioxidant activity of extracts [51]. Owing to the many processes underlying antioxidant action, no standardized approach exists to thoroughly assess the antioxidant activity of substances [52]. Consequently, we employed DPPH to assess the antioxidant activity of the MGSP-chicken myofibrillar protein complex. In Figure 6, the antioxidant activity of the groups supplemented with MGSP exhibited an increase. The MGSP-2 group had the most significant gain, rising by 27.35%. The presence of rich phenolic compounds in grape seed powder treated with an alternating-current electric field may account for this [48]. Phenols and proteins can form complexes via noncovalent or covalent interactions, which enhances antioxidant ability and protects phenols from degradation [52]. The interaction between protein and phenol influences gel network formation and enhances water retention [53], aligning with the improved water retention depicted in Figure 1. However, when the addition surpassed 4%, DPPH exhibited a little decline. The presence of excessive particles may interfere with the formation of the chicken protein gel network, resulting in a loose structure that exposes some lipids to oxygen, hence facilitating oxidation and reducing some antioxidant capabilities.

### 3.9. Intermolecular Forces

**Figure 7:**
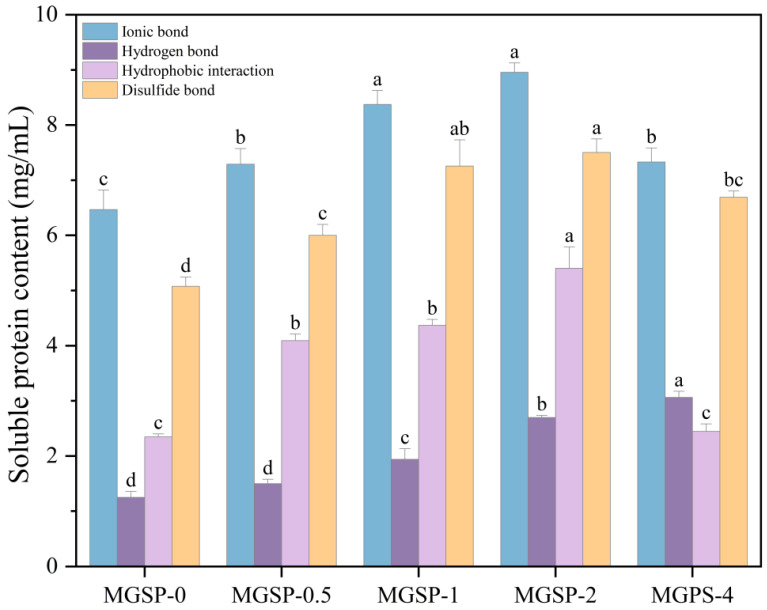
The influence of MGSP addition amount on the intermolecular forces of MP gels. a–d: Distinct lowercase letters signify substantial changes in values among groups (*p* < 0.05). Error bars indicate the standard deviation.

The intermolecular forces are intrinsically linked to the characteristics of gels. Hydrophobic interactions and covalent bonds sustain protein connections, whereas hydrogen bonds and ionic bonds govern the interactions between proteins and water [44]. The intermolecular pressures were evaluated based on the soluble protein content, as depicted in Figure 7. Ionic and disulphide bonds are the primary determinants that stabilize the gel structure. The incorporation of MGSP amplified the magnitude of the four forces, suggesting that the spatial configuration of the protein may alter as a result of the interactions among the proteins [54]. Ionic bonds are reinforced at low concentrations, potentially due to the intensified non-covalent interactions between MGSP (negatively charged) and the positively charged amino acid residues on the protein surface. However, excessive concentration may induce an electrostatic shielding effect, thereby somewhat diminishing the formation of ionic bonds [55]. The enhancement can be ascribed to the abundance of phenolic compounds in MGSP. The hydroxyl groups of these compounds promote the establishment of hydrogen bonds between proteins and water molecules [39]. Research indicates that hydrogen bond formation can strengthen the binding affinity between proteins and polyphenols [56]. The enhancement of hydrophobic association indicates that, during thermal gelation, the combination of MP gels and MGSP promotes the development of the protein structure, hence strengthening hydrophobic interaction. When the ratio surpasses 4%, this reduction may result from elevated MGSP enhancing water absorption, thereby limiting the expansion of the protein structure and the accessibility of hydrophobic groups. This resembles the phenomena documented by Jiang et al. [57] on the incorporation of spun soy protein into MP. The substantial rise in disulphide bonds may result from the presence of polyphenols in MGSP, which are susceptible to oxidation to quinones during gel heating and exhibit strong reactivity with free sulfhydryl groups, thus facilitating disulphide bond formation in proteins [39]. The assertion in the preceding text that the quantity of free sulfhydryl groups has diminished corroborates this claim. The augmentation of disulphide linkages can render the protein structure more inflexible, aligning with the previously noted enhancement in gel strength. In conclusion, the application of MGSP will influence intermolecular forces and facilitate the formation of a more stable gel network structure.

### 3.10. Rheological Characteristics

**Figure 8:**
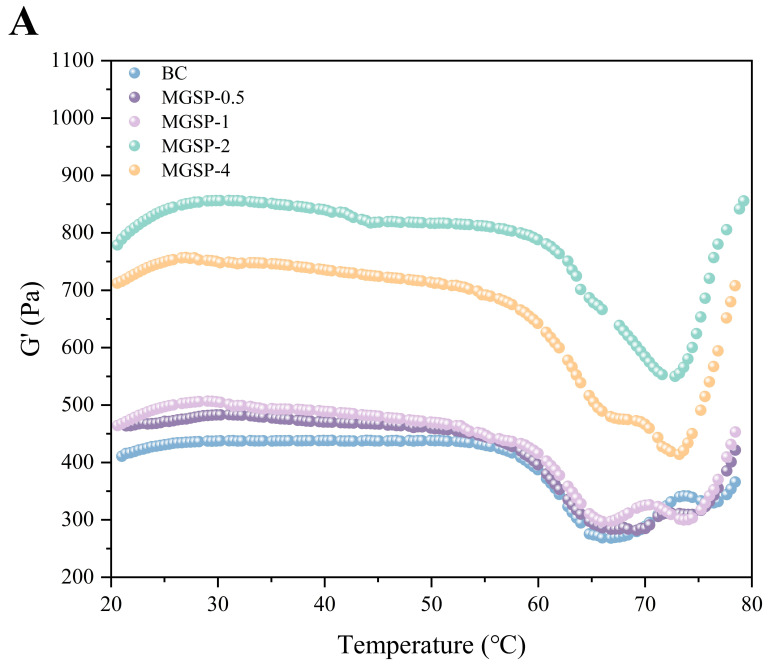

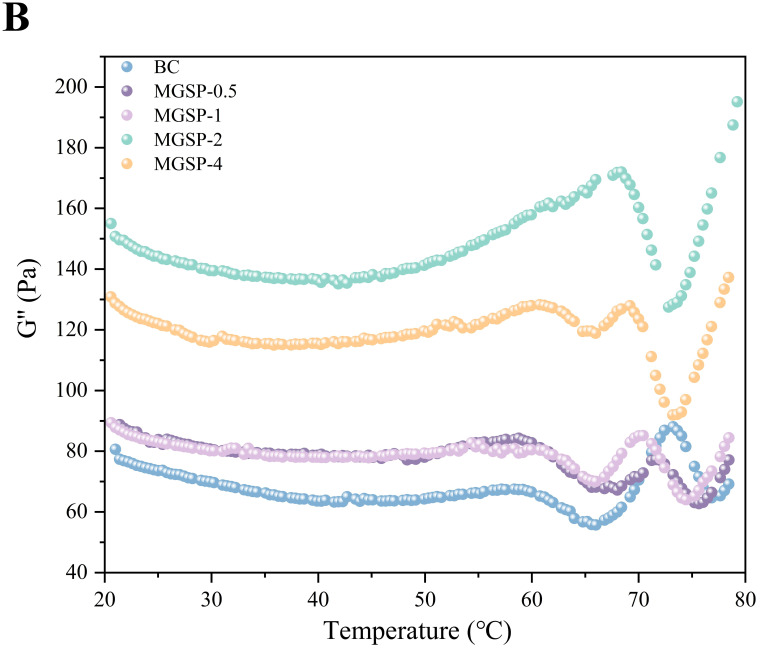
The impact of MGSP on the alterations of the G’ (**A**) and G” (**B**) curves throughout the thermal treatment of the MP gel.

The production of MP gels under varying quantities of MGSP conditions was examined using dynamic oscillatory rheological analysis (Figure 8). The curves of the five sets of MP gels exhibited a comparable tendency. From a rheological standpoint, G’ denotes the elastic qualities of the material, whereas G” signifies its viscous characteristics [58]. G’ significantly exceeds G”, indicating that the MP gels possess elastic characteristics. The trends of G’ and G” are consistent. Furthermore, the figure illustrates a substantial increase in the elasticity of the MP gel containing MGSP. Throughout the heating procedure from 20 to 80 °C, G’ exhibited five distinct stages. During the initial phase (20–50 °C), G’ exhibited stability, signifying that the system was transitioning from a solution to a gel [59]. During the second stage (50–65 °C), G’ progressively diminished, attaining its minimum value at 65 °C. This may result from the initiation of denaturation and unfolding of the myosin heads, which disrupted the non-covalent connections maintaining the network structure [60], leading to a decrease in the orderliness of protein molecules and, consequently, a decline in G’. During the third stage (65–70 °C), G’ attained a low before subsequently rising, presumably due to the unfolding of myosin tails, which exposed various hydrophobic areas and sulfhydryl groups. This resulted in the assembly of a unique protein network framework via hydrophobic interactions and the establishment of disulphide bonds [61]. The active chemicals, such as polyphenols, in MGSP may attach to protein molecules via hydrophobic interactions, thereby reinforcing the network structure [62]. During the fourth stage (70–75 °C), G’ saw a minor decline, likely attributable to the degradation of certain unstable non-covalent cross-links established in the preceding stage due to elevated temperatures [63]. The concluding phase (75–80 °C) exhibited a significant rise in G’, marking the completion of gel network development. The exposed sulfhydryl groups underwent significant oxidation, resulting in the formation of stable disulphide bonds, while hydrophobic contacts progressively intensified, leading to increased protein contraction and aggregation into a highly cross-linked, stable gel network [64].

The fluctuation of “G” is illustrated in Figure 8B. The trajectory of the G” curve parallels that of G’. The initial peak temperature of the MP gels with included MGSP migrated to the left. This suggests that the implementation of MGSP has facilitated the formation of disulphide and hydrophobic linkages, hence enhancing the crosslinking between MP and proteins, as corroborated by Zhang et al. [65]. Consequently, the incorporation of MGSP can facilitate the development of the gel network by lowering the denaturation temperature at the MP terminus, thereby enhancing the network architecture.

### 3.11. FTIR

**Figure 9:**
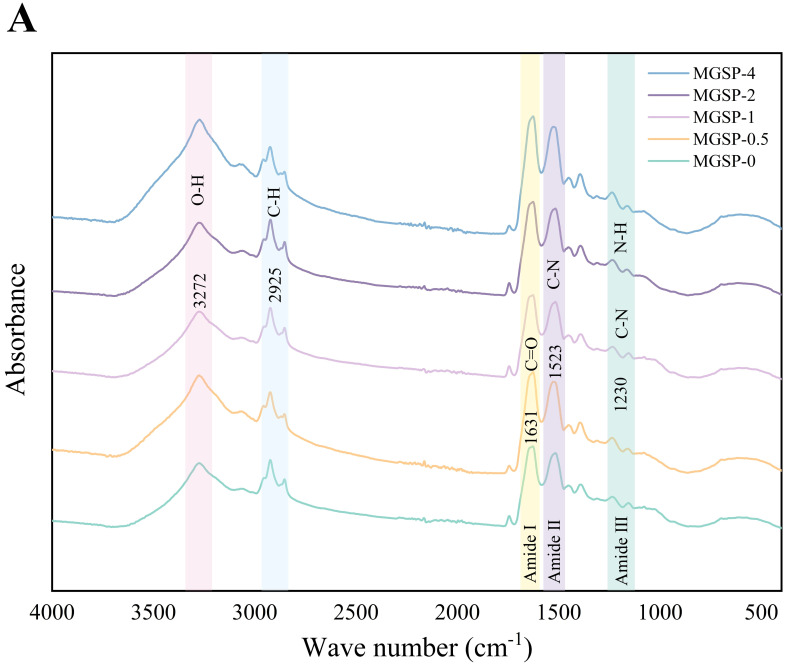

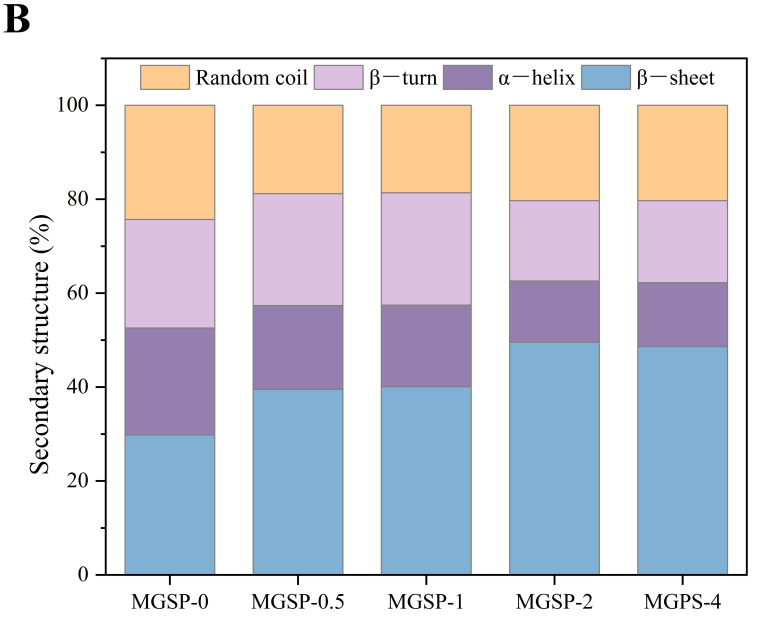
The Fourier spectrograms of all the samples. (**A**) Infrared spectrum, (**B**) proportion of protein secondary structure.

FTIR is an efficient technique for analyzing protein structure. Various chemical bonds or functional groups inside protein molecules absorb infrared light distinctly and exhibit differing vibrational frequencies, therefore indicating the secondary structure of proteins [66]. The FTIR spectra results of chicken myofibrillar protein gel depicted in Figure 9A demonstrates that the addition of MGSP does not produce new peaks, signifying that no new covalent connections are established between MP and MGSP; rather, they are associated by non-covalent interactions. The extensive absorption peak at 3272 cm^−1^ corresponds to amide A (3600–3200 cm^−1^), primarily attributed to O-H stretching vibrations. The peak strength often rises upon the addition of MGSP, likely attributable to enhanced absorption signals from phenolic compounds in MGSP, suggesting that MGSP’s presence may augment hydrogen-bond content [67]. The peak at 2925 cm^−1^ corresponds to amide B (3000–2800 cm^−1^), mostly associated with the antisymmetric stretching of -CH_2_ groups. The peak at 1631 cm^−1^ arises from the C=O stretching vibration in the protein backbone and is part of the amide I region (1680–1630 cm^−1^). The N-H planar bending and C-N stretching vibrations at 1523 cm^−1^ align with the amide II area (1570–1510 cm^−1^). The C-H stretching vibration at 1230 cm^−1^ pertains to the amide III area (1350–1200 cm^−1^) [68]. The intensity of the amide I peak augmented, whilst the amide II region of the composite gel exhibited a minor red shift. This may be ascribed to the electrostatic interaction between MP and MGSP [65]. The amide I band (1680–1630 cm^−1^) predominantly encompasses the β-sheet region (1640–1600 cm^−1^), the random coil region (1650–1640 cm^−1^), the α-helix region (1660–1650 cm^−1^), and the β-turn region (1700–1660 cm^−1^) [69], which is intricately associated with alterations in protein secondary structure.

To assess the impact of varying dosages of MGSP on protein structural alterations, the percentage of secondary structure within the amide I band was determined (Figure 9B). MGSP can influence the secondary structure of proteins, with β-structures predominating. The α-helix content in the blank gel exceeds that in the gel containing MGSP. MGSP-4 diminished the content from 22.78% (control group) to 13.06%, reflecting a maximum reduction of 42.66%. Conversely, the β-sheet content rose compared to the control group, with a peak increase of 66.23% (MGSP-4). This outcome demonstrates that the incorporation of MGSP facilitated the unwinding of the α-helix configuration of myofibrillar proteins, converting them into a β-sheet structure and enhancing intermolecular interactions and conformation. This may result from the interaction between the fibers and proteins. This process produces a specific protein, consequently augmenting the quantity of β-sheet chains and the β-angle, and facilitating the development of a denser gel structure [70]. Furthermore, pertinent research suggests that the dissociation of α-helices and the establishment of β-sheet configurations facilitate myosin gelation [71].

### 3.12. SEM

**Figure 10:**
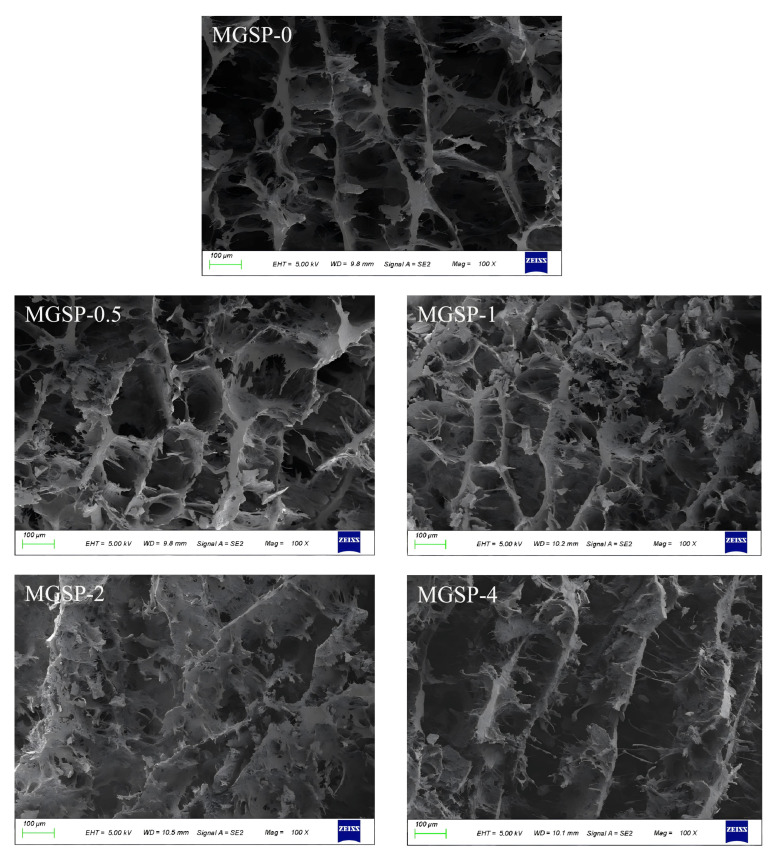
The microstructure of MP gels × 100 magnification.

Figure 10 presents the microstructure diagrams (SEM) of different gels. The implementation of MGSP has modified the composition of the protein gel. The gel devoid of MGSP addition displays a conventional porous network structure; however, the pore distribution is irregular and the structure is notably loose, perhaps resulting in inadequate stability. Conversely, the composite gel demonstrates a denser and more uniform porosity architecture, particularly at an addition of 2%. The incorporation of MGSP occupies gaps in the gel matrix, enhances gel rigidity, and fortifies the network, rendering it more cohesive. The alternating electric field can improve the dispersibility and surface activity of grape seed powder, therefore augmenting its specific surface area [72]. This structural alteration may improve the material’s capacity to absorb water [73], consequently reinforcing protein interactions through non-covalent forces and facilitating gel network development. At elevated concentrations (4%), the interprotein distance within the gel system expands, resulting in enlarged porous structures. The research findings indicate that an excessive incorporation of insoluble dietary fiber enlarges the separation between protein molecules and obstructs protein cross-linking [74]. Nevertheless, the overall structure of MGSP-4 exceeds that of the control group. This indicates that the alternating current field acts as an efficient modification method to augment the functional properties of grape seed powder and boost the structural integrity of the MP gel.

## 4. Conclusions

This research methodically examined the impact of MGSP on the structure and functionality of chicken myofibrillar protein gels. Gels with varying properties (0%, 0.5%, 1%, 2%, and 4%) were produced by modifying the MGSP addition ratio. The findings indicate that MGSP can augment the water retention and solubility of the MP gel, while also enhancing gel strength. The free-thiol and carbonyl concentrations, along with DPPH data, demonstrated an enhancement in the antioxidant activity of the MP gel. The tests on intermolecular forces revealed that ionic and disulphide bonds were the predominant forces in the MP gel and that the addition of MGSP might augment interactions among protein molecules. The secondary structure study indicated that the incorporation of MGSP would disrupt the α-helical configuration and convert it into a β-sheet, thus altering the protein’s conformation. SEM demonstrated an enhanced microstructural configuration of the MP gel. The optimal gel properties were seen with the addition of 2% to 4%. This experiment possesses specific restrictions. We exclusively assessed the physical, chemical, and structural aspects. Subsequent research should explore additional aspects, encompassing sensory attributes, digestive properties, and storage efficacy. This study provides valuable theoretical information for enhancing the quantity and quality of meat products in chicken production. The findings elucidate the interaction between modified grape seed powder and myofibrillar protein, contributing to the development of functional plant-based ingredients to improve the quality of protein gel systems.

## Figures and Tables

**Table 1:** The color difference and whiteness of chicken MP.

Samples	L*	a*	b*	*W*
MGSP-0	86.57 ± 0.16 ^a^	0.84 ± 0.01 ^e^	0.51 ± 0.03 ^a^	86.88 ± 0.71 ^a^
MGSP-0.5	84.45 ± 0.17 ^b^	1.27 ± 0.03 ^d^	0.47 ± 0.01 ^b^	84.39 ± 0.17 ^b^
MGSP-1	83.93 ± 0.06 ^c^	1.43 ± 0.03 ^c^	0.37 ± 0.02 ^c^	83.90 ± 0.01 ^b^
MGSP-2	82.77 ± 0.06 ^d^	1.64 ± 0.03 ^b^	0.26 ± 0.03 ^d^	82.69 ± 0.06 ^c^
MGSP-4	81.82 ± 0.08 ^e^	1.71 ± 0.01 ^a^	0.20 ± 0.02 ^e^	81.74 ± 0.08 ^d^

a–e: This signifies substantial disparities among several letters within the same column of samples (*p* < 0.05). The values are shown as the mean ± standard deviation.

**Table 2:** The effect of different contents of MGSP on the TPA of myofibrillar protein gel.

Samples	Hardness (g)	Springiness (g)	Cohesiveness (g)	Gumminess (g)
MGSP-0	222.80 ± 6.31 ^d^	0.82 ± 0.01 ^a^	0.59 ± 0.01 ^a^	117.49 ± 4.66 ^d^
MGSP-0.5	229.84 ± 5.77 ^d^	0.80 ± 0.02 ^a^	0.54 ± 0.01 ^b^	137.61 ± 5.65 ^c^
MGSP-1	281.60 ± 8.45 ^c^	0.79 ± 0.02 ^a^	0.49 ± 0.01 ^c^	167.53 ± 4.37 ^b^
MGSP-2	342.86 ± 4.45 ^a^	0.73 ± 0.01 ^b^	0.34 ± 0.02 ^d^	209.40 ± 5.95 ^a^
MGSP-4	325.41 ± 3.19 ^b^	0.69 ± 0.02 ^b^	0.28 ± 0.02 ^e^	175.81 ± 2.76 ^b^

a–e: This signifies substantial disparities among several letters within the same column of samples (*p* < 0.05). The values are shown as the mean ± standard deviation.

## Data Availability

The original contributions presented in the study are included in the article; further enquiries can be directed to the corresponding author.
